# Aberrant Expression of Folate Metabolism Enzymes and Its Diagnosis and Survival Prediction in Ovarian Carcinoma

**DOI:** 10.1155/2019/1438628

**Published:** 2019-03-31

**Authors:** Jia Chen, Li Li

**Affiliations:** ^1^Department of Radiology, Affiliated Tumor Hospital of Guangxi Medical University, Nanning, Guangxi, China; ^2^Department of Gynecologic Oncology, Affiliated Tumor Hospital of Guangxi Medical University, Nanning, Guangxi, China; ^3^Laboratory of Early Prevention and Treatment for Regional High Frequency Tumor, Ministry of Education Key Laboratory, Nanning, Guangxi, China

## Abstract

This study was to validate changes in the levels of folate receptor-*α* (FOLR1), dihydrofolate reductase (DHFR), and methionine synthase reductase (MTRR) in the tissue of OC patients. The expression of FOLR1, DHFR, and MTRR was evaluated in 80 cases of primary OC, 50 cases of benign ovarian tumors, and 30 normal ovarian tissues. Associations between protein expression and clinicopathological characters were assessed, and diagnostic and prognostic evaluation of FOLR1, DHFR, and MTRR was performed. Results showed that upregulated FOLR1 and MTRR and downregulated DHFR were detected in OC. Patients with abnormality of FOLR1, DHFR, and MTRR tend to have a higher percentage of platinum resistance. Moreover, the areas under receiver operating characteristic curves (AUCs-ROC) for FOLR1, DHFR, and MTRR were 0.723, 0.717, and 0.714, respectively. The combination of FOLR1, DHFR, and MTRR could produce an area of 0.864 under the receiver-operating characteristic curve in distinguishing platinum-resistant patients from platinum-sensitive patients (*P* < 0.0001). Correlations were present between the expression of FOLR1, DHFR, and MTRR. Furthermore, Kaplan-Meier curves indicated that the patients with overexpressed MTRR had a poorer overall survival time compared to those with low expression (*P* < 0.05). Thus, folate metabolic enzymes could provide a potential promising biomarker for diagnosis platinum-resistant in OC.

## 1. Introduction

Ovarian cancer is the fourth most common cancer in women worldwide, and it has the most noteworthy lethal rate around gynecologic malignancies. Two most critical barriers to treatment of ovarian malignancy are absence of early diagnostic markers and advancement of drug resistance after therapy, especially in advanced stages. Various epigenetic changes have been recognized in ovarian cancer. Recent progresses in our understanding of molecular pathogenesis of ovarian malignancy have dramatically provided potential new targets for molecularly targeted therapies. There thus is a critical need for improved biological markers and therapies for ovarian carcinoma, which will come from a better understanding of the biology of the disease.

Folate is an essential component in DNA synthesis, replication and repair, protein synthesis, and methylation reactions. This is especially true for rapidly dividing cells [1]. Folate receptor 1 (FOLR1) internalizes folates by means of receptor-mediated endocytosis and reduced folate carrier (RFC) uses a bidirectional anion-exchange mechanism to transport folates into cytoplasm [2, 3]. Dihydrofolate reductase (DHFR) catalyzes the reduction of dihydrofolate (DHF) to tetrahydrofolate (THF), which plays a vital role in cellular metabolism and cell growth [4]. Methionine synthase reductase (MTRR) is an enzyme controlling the activity of MTR in folate metabolism by transferring the methyl group of methyltetrahydrofolate to homocysteine via the methionine synthase, which is responsible for DNA methylation [5].

In the previous research in our lab, ovarian cancer-resistant cell lines were established to screening drug-resistant genes [6]. Most of them are associated with metabolism, especially folate metabolism. Meanwhile, we have reported that overexpression of MTRR plays an important role in cisplatin resistance, and silencing MTRR expression partially reverses cisplatin-resistant phenotype [7]. In the present study, the expression levels of FOLR1, DHFR, and MTRR were examined in OC tissues, and the prognostic ability of these three proteins was investigated and compared.

The aim of this study was to validate changes in the levels of FOLR, DHFR, and MTRR in the tissue of OC patients. We sought to determine whether folate metabolism enzymes could serve as a novel biomarker for early diagnosis and prognosis of platinum-resistant OC patients, as well as their clinical significance in OC.

## 2. Materials and Methods

### 2.1. Clinical Samples and Follow-Up

OC tissues and normal ovary tissues were collected from patients who were treated in the Department of Gynecologic Oncology of the Affiliated Tumor Hospital of Guangxi Medical University between 2004 and 2010. All the patients were pathologically diagnosed with OC. Pathological stage and histological subtype were determined according to the International Federation of Gynecology and Obstetrics (FIGO) criteria and the World Health Organization criteria. Clinical and pathological data was collected from the medical records including age, surgical stage, metastasis, ascites, tumor grade and subtype, and drug resistance. Samples were collected from 80 cases of primary OC, 50 cases of benign ovarian tumors, and 30 normal ovarian tissues. The median age was 41.1 years (range: 13-76 years) in the OC group, 40.1 years (range: 10-74 years) in the benign ovarian tumor group, and 43.1 years (range: 29-60 years) in the normal ovary group. The 80 OC patients underwent surgical intervention for OC of whom 61 patients with epithelial ovarian cancer received chemotherapy with cisplatin plus paclitaxel and 19 patients with nonepithelial ovarian cancer were treated with cisplatin, bleomycin, and vincristine. The study was approved by the Ethics Committee of Guangxi Medical University. Written informed consent was obtained from all the subjects before study.

### 2.2. Western Blotting Analysis

160 fresh specimens were sonicated with an ultrasonic tissue disrupter in lysis buffer for 30 min. The tissue debris was pelleted by centrifugation, and supernatants were collected. After measuring the protein concentration by BCA protein assay, proteins were subjected to SDS-PAGE and then transferred onto PVDF membranes. After blocking, the membranes was treated with 5% (*w*/*v*) BSA in PBST (PBS, pH 7.5, containing 0.1% Tween-20) and then incubated with primary antibodies MTRR (1 : 1000; Santa Cruz Biotechnology), FOLR1 (1 : 1200; Abcam), and DHFR (1 : 1000; Santa Cruz Biotechnology) overnight at 4°C. These PVDF membranes were subsequently treated with PBST and incubated with peroxidase-conjugated secondary antibody (1 : 1000) (Santa Cruz Biotechnology) for 1 h. Visualization was detected by using a chemiluminescence system (Pierce, USA) according to the manufacturer's instructions. The band intensities were quantified using the ImageQuant software (Molecular Dynamics, Sunnyvale, CA, USA). Then, the membranes were stripped and reincubated with anti-GAPDH (1 : 1000; Santa Cruz Biotechnology) for normalization.

### 2.3. Immunohistochemistry

Paraffin-embedded sections (5 *μ*m) of ovarian tissues were obtained, deparaffinized, and rehydrated through a graded ethanol series. Antigen retrieval was done in 10 mM citrate buffer (pH 6.0) at 120°C for 2 min. The sections were allowed to cool to 30°C and washed with phosphate-buffered saline (PBS, pH 7.3). After inactivating the endogenous peroxidase with 3% H_2_O_2_ for 10 min and washing with PBS, sections were incubated at 4°C overnight with primary antibodies in PBS and then washed with PBS. The primary antibodies used were polyclonal MTRR antibody (1 : 200; sc-48889, Santa Cruz Biotechnology), polyclonal FOLR1 antibody (1 : 200; ab-3361, Abcam), and polyclonal DHFR antibody (1 : 200; sc-14778; Santa Cruz Biotechnology). Sections were stained with an ultrasensitive streptavidin-peroxidase kit (Maixin Bio, Kit 9719, Fuzhou, China), and visualization was performed with 3,3′-diaminobenzidine (DAB). Nuclei were stained with Harris Hematoxylin (Sairuida.Bio, Tianjin, China). In negative control, the primary antibody was replaced with PBS. A colon cancer sample was used as a positive control. Positive cells had brown granules in the cytoplasm. The positive cancer cells were semiquantitatively determined based on the staining intensity and percentage of positive cells. Sections were scored based on the chromatic intensity: 0, no pigmentation; 1, light yellow; 2, buff; and 3, brown. Five fields were randomly selected from each section, and the mean percentage of positive cells was determined: 0, <5%; 1, 5%-25%; 2, 26%-50%; 3, 51%-75%; and 4, *d* > 75%. The immunohistochemical scores were multiplied by the intensity score and percentage of positive cells: 0-2 (-); 3-4 (+); 5-8 (++); and 9-12 (+++). The sections were independently assessed by two observers.

### 2.4. Statistical Analysis

Nominal variables were compared using the *χ*^2^ test, and ordinal categorical variables were evaluated by a nonparametric Spearman's rank test. Receiver-operating characteristic (ROC) curves were established to evaluate the diagnostic value of FOLR1, MTRR, and DHFR for differentiating benign and malignant, and the cutoff values were also calculated. According to the cutoff value of the ROC curve, we defined the result of protein below the cutoff value as low expression and above the cutoff value as high expression. OS curves were plotted by the Kaplan-Meier method and compared by log-rank test. The assessment of correlation between survival time and multiple clinicopathological variables was carried out by the Cox proportional hazards regression model. Univariate and multivariate Cox proportional hazard models were used to identify variables associated with OS in the group of OC patients. When the significant variables associated with OS were obtained by univariate analysis, multivariate analysis was used for evaluating which variables were the most important in prediction of OS. A *P* value less than 0.05 was considered as statistically significant. All of the statistical calculations were performed using the SPSS software (19.0, Chicago, IL, USA), and GraphPad Prism 5.0 (GraphPad Software Inc., CA) was used to generate graphs. All of the *P* values <0.05 were considered to be statistically significant.

## 3. Results

### 3.1. Expression of FOLR, DHFR, and MTRR

**Figure 1:**
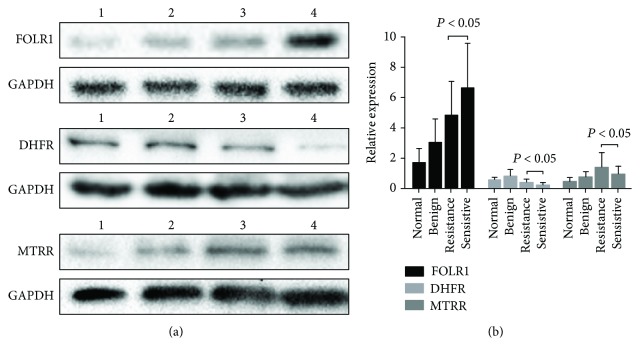
(a) Western blot assay of FOLR, DHFR, and MTRR expression in different ovarian tissues. Lane 1: normal ovarian tissues; lane 2: benign ovarian tumor; lane 3: platinum-resistant OC; and lane 4: platinum-sensitive OC. (b) Quantitative analysis of FOLR, DHFR, and MTRR expression in normal ovarian tissues and OC. FOLR, DHFR, and MTRR expression in Western blot assay is expressed as the ratio of OD of FOLR, DHFR, and MTRR to that of GAPDH. Data are expressed as means ± standard error (SEM).

FOLR, DHFR, and MTRR expression in tissues of 160 patients was assessed by Western blot analysis (Figure 1). Quantitative analysis of Western blotting analysis showed that FOLR1 and MTRR have the highest expression in OC tissues, while DHFR has the highest expression in benign tumor tissues (Figure 1(a)). DHFR and MTRR expression was clearly elevated in platinum-resistant OC compared with platinum-sensitive OC, while FOLR1 expression in platinum-resistant OC was lower than in platinum-sensitive OC (Figure 1(b)). The correlation between the expression of FOLR, DHFR, MTRR and the kinds of ovarian tissue is summarized in Table 1. The expression of FOLR, DHFR, and MTRR was significantly correlated with the kinds of ovarian tissue (*P* < 0.05, respectively).

**Figure 2:**
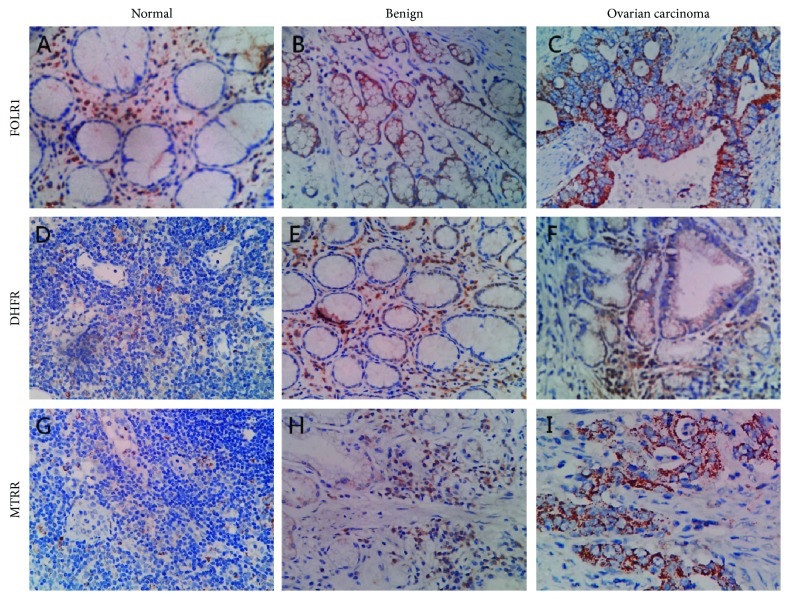
Immunohistochemical results of FOLR, DHFR, and MTRR in different ovarian tissues. (a–c) FOLR1 expression in different ovarian tissues. (d–f) DHFR expression in different ovarian tissues. (g–i) MTRR expression in different ovarian tissues (original magnification, 200x).

In order to detect the location, FOLR, DHFR, and MTRR expression in clinical samples was assessed by immunohistochemical staining of sections isolated from 10 OC, 10 patients with benign tumors OC, and 10 patients with normal ovaries. Representative examples of staining are shown in Figure 2. Immunohistochemistry showed that FOLR-, DHFR-, and MTRR-positive cells had brown granules in the cytoplasm. FOLR1 and MTRR had moderate to strong expression in OC tissues, whereas DHFR demonstrated little or no immunoreactivity in OC tissues (data not shown).

### 3.2. Expression of FOLR, DHFR, and MTRR in Ovarian Samples Correlates with Clinicopathological Features

The correlations of the expression of FOLR, DHFR, of MTRR with various clinical variables are listed in Table 2. The results showed that the expression of the FOLR and MTRR proteins did not correlate with omentum metastasis and ascites. However, the expression of DHFR was significantly correlated with the omentum metastasis and outcome (*P* < 0.05, respectively).

### 3.3. The Diagnostic Efficacy of FOLR, DHFR, and MTRR

**Figure 3:**
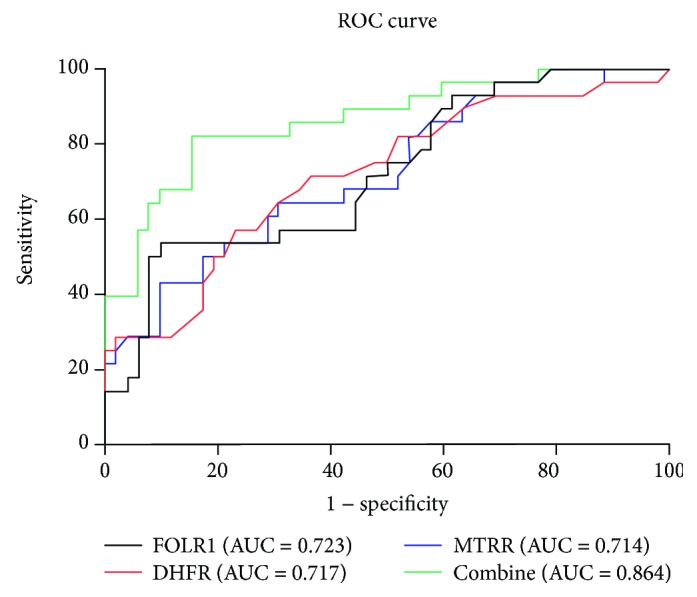
ROC curves for FOLR, DHFR, and MTRR and the combination of the three enzymes in predicting platinum resistance.

ROC curve analyses were performed to evaluate the diagnostic accuracy of the FOLR, DHFR, and MTRR. ROC curve analyses revealed that when the optimal cutoff values of FOLR, DHFR, and MTRR were 3.855, 0.185, and 1.425, respectively, the area under the curve (AUC) values for them were 0.723 (95% CI: 0.606-0.840, *P* < 0.001; sensitivity = 88.20%, specificity = 55.20%), 0.717 (95% CI: 0.597-0.837, *P* < 0.001; sensitivity = 69.00%, specificity = 62.70%), and 0.714 (95% CI: 0.594-0.833, *P* < 0.001; sensitivity = 44.80%, specificity = 92.20%), respectively (Figure 3). In the next step, we further explored whether the combination of FOLR, DHFR, and MTRR significantly improved the diagnostic efficiency (AUC 0.864, 95% CI: 0.777–0.951, *P* < 0.0001; Figure 3). The sensitivity, specificity, and accuracy of FOLR, DHFR, and MTRR and the combination (FOLR+DHFR+MTRR) for distinguishing platinum-resistant OC patients from platinum-sensitive controls are summarized in Table 3. The results showed that the combination group had higher sensitivity and specificity. Together, these results indicated that protein FOLR, DHFR, and MTRR had potential significance with respect to the sensitivity and specificity in the diagnosis of platinum-resistant OC.

### 3.4. Correlation between the Expression of FOLR, DHFR, and MTRR

FOLR, DHFR, and MTRR were located and coexpressed in the cytoplasm of the OC tissue (Figure 2). The correlation analysis between the expression of FOLR, DHFR, and MTRR in the OC tissues is summarized in Table 4. The results showed that positive MTRR expression was significantly associated with positive FOLR1 and DHFR expression (*P* < 0.001 and <0.01, respectively). Correlation was also found between positive FOLR1 and positive DHFR (*P* < 0.05).

### 3.5. Cox Proportional Hazard Regression Models of Risk Factors Associated with OS among OC Patients

The results of univariate and multivariate analysis are presented in Table 5. Histology, node status, omentum metastasis, organ metastasis, FOLR1, and DHFR are not significant predictive factors for the prognosis of OC patients as determined by univariate analysis (*P* > 0.05). However, FIGO Stage, grade, ascites, platinum resistance, and high expression of MTRR were significant predictive factors for the prognosis of OC patients (*P* < 0.05 for all, Table 5). In the multivariate Cox analysis of OS, FIGO Stage, grade, ascites, platinum resistance, and high expression of MTRR were not independent predictive risk factors for the prognosis of OC patients (*P* > 0.05).

### 3.6. Survival Analysis and Prognostic Significance of the Expression of FOLR, DHFR, and MTRR

**Figure 4:**
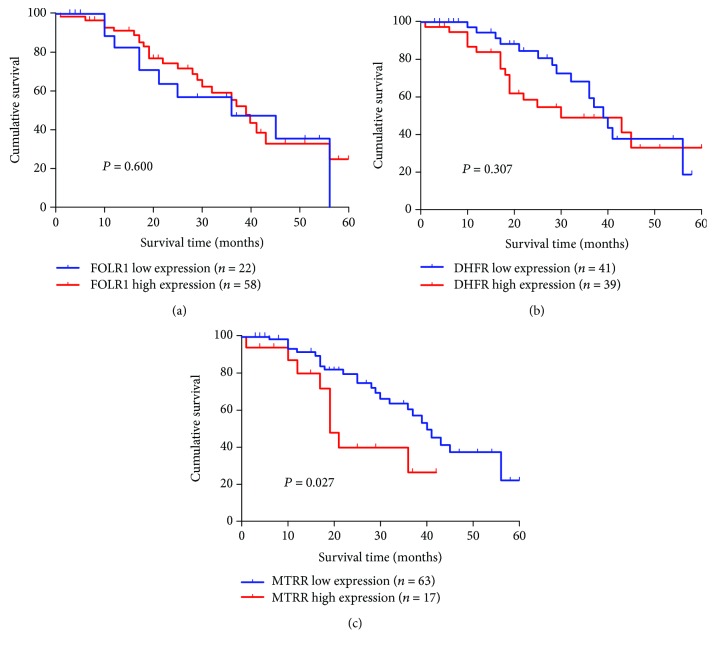
Survival analysis of 80 OC patients by the Kaplan–Meier method. (a) FOLR1 for survival analysis of OC patients; (b) DHFR for survival analysis of OC patients; (c) MTRR for survival analysis of OC patients. Patients with FOLR, DHFR, and MTRR expression higher or lower than average expression are considered as high or low, respectively.

To establish survival curves, continuous expression levels of FOLR1, DHFR, and MTRR were converted to a dichotomous variable, using their cutoff values from ROC curve analyses as a threshold, respectively. The Kaplan-Meier method was employed to analyze the OS times of 80 OC patients between high expression and low expression of FOLR, DHFR, and MTRR. As shown in Figures 4(a) and 4(b), OC patients with different OS times could not be distinguished by FOLR1 or DHFR alone (*P* > 0.05). However, MTRR was more sensitive for predicting prognosis in subgroups of OC patients (*P* < 0.01, Figure 4(c)). Moreover, the median survival time of OC patients with low (*n* = 63) and high (*n* = 17) levels of MTRR was 40 and 19 months, respectively.

## 4. Discussion

**Figure 5:**
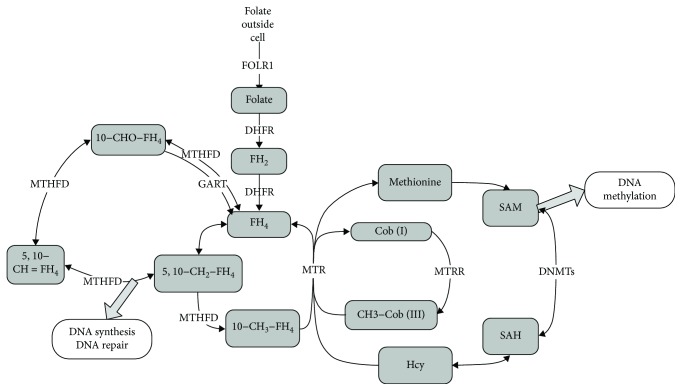
Schematic overview of the folate metabolic pathway mediated by FOLR, DHFR, and MTRR. Arrows that cross the line indicate metabolites that can travel between compartments. FOLR1: folate receptor 1; DHFR: dihydrofolate reductase; MTRR: methionine synthase reductase; MTR: methionine synthase; FH4: tetrahydrofolate; 5,10-CH2-FH4: 5,10-methylenetetrahydrofolate; SAM: S-adenosylmethionine; SAH: S-adenosylhomocysteine; Hcy; homocysteine; MTHFD: methylenetetrahydrofolate dehydrogenase; GART: phosphoribosylglycinamide formyltransferase; DNMTs: DNA methyltransferases.

The folate metabolic pathway comprises a cycle mediated by FOLR1, DHFR, and MTRR (Figure 5). Aberrant promoter methylation has been linked to the development of OC poor prognosis or clinical severity in several cancer types, including platinum resistance [8–10]. Our results indicated that the abnormal folate metabolic pathway could offer advantages for platinum resistance and proliferation in OC.

FOLR1 overexpresses in a wide range of epithelial malignant cancers [11–14]. Some studies have developed that serum FOLR1 is a biomarker for ovarian cancer with implications for diagnosis, prognosis, and prediction of OC [11, 15, 16]. Song et al. demonstrated that suppression of FOLR1 reversed taxol resistance in nasopharyngeal carcinoma cell lines [17]. Moreover, in neuroendocrine tumor, low FOLR1 expression was also identified as a marker for more aggression and associated with shorter OS and PFS [18]. In this study, we found that FOLR1 was upregulated in OC tissues compared with the normal ovarian tissues and benign ovarian tumor as well. However, high levels of FOLR1 were not associated with shorter OS of OC patients.

Interfering with the expression of DHFR is an approach in improving pharmacokinetics and reversing drug resistance in OC [19, 20]. In NSCLC, osteosarcoma, and lymphoblastic leukemia, DHFR expression or polymorphism also has been associated with sensitivity to drug resistance [21–24]. Additionally, non-small cell lung carcinoma patients with low DHFR expression had a longer median PFS and OS compared with patients with a higher DHFR expression. However, the difference was not statistically significant [25]. Our data also demonstrated that overexpression of DHFR is associated with platinum resistance in OC. Similarly, overexpression of DHFR was not associated with shorter OS of OC patients.

MTRR (rs1801394) has been linked to many cancers [26–28]. But early studies reported no significant association between polymorphisms in the MTRR genes and ovarian cancer risk [29–31]. In our previous study [7], we found that the overexpression of MTRR is related to cisplatin resistance, most probably because of inducing apoptosis and reducing autophagy in OC cells. In this study, MTRR's actual impact on clinical outcome of OC patients is still very scarce and incomplete. In this study, we confirmed the presence of increased MTRR expression in the majority of our platinum-resistant patients. The OS difference was found between patients with high and low MTRR expression.

Our results showed that the combined ROC analysis revealed an AUC value of 0.832 in discriminating platinum-resistant patients from platinum-sensitive patients. This analysis provided information for an effective combined prognostic approach for drug resistance. To our knowledge, this is the first report showing the relationship between platinum resistance and expressions of the three folate metabolism enzymes in OC.

The limitations of this study need to be presented. First is the lack of other types of histology disease and a small sample size of OC patients, which could lead to the lack of power and the consequent imprecision. Second, although the biological functions of the three folate metabolism enzymes have been inferred by previous gene functional analysis, the mechanisms behind the predictive values of these three folate metabolism enzymes in OC are still not clear, and their functional roles should be further explored in experimental studies.

In summary, the evaluation of FOLR1, DHFR, and MTRR expression may provide useful information for doctors to make optimal clinical decisions. MTRR is a better factor for determining the prognosis of OC. Combining the three enzymes diagnosis might contribute to the identification of patients who are likely to develop chemotherapy resistance and may be a novel potential target for OC therapy, which will require analysis by further validation studies.

## Figures and Tables

**Table 1:** FOLR1, DHFR, and MTRR expression in different tissues.

Groups	Total	FOLR1		DHFR		MTRR	
		Expression	*P* value	Expression	*P* value	Expression	*P* value
Malignant	80	5.951 ± 0.321	0.001	0.244 ± 0.026	0.001	1.044 ± 0.088	0.001
Benign	50	3.003 ± 0.222		0.776 ± 0.071		0.687 ± 0.059	
Normal	30	1.663 ± 0.181		0.532 ± 0.041		0.432 ± 0.055	

**Table 2:** Correlation between FOLR1, DHFR, and MTRR expression and clinicopathological parameters.

Parameters		Total	FOLR expression	*P*	DHFR expression	*P*	MTRR expression	*P*
Histology	Epithelial	61	4.979 ± 2.326	0.001	0.234 ± 0.227	0.543	0.872 ± 0.752	0.001
	Other	19	8.682 ± 2.501		0.271 ± 0.261		1.528 ± 0.697	

FIGO Stage	I-II	37	4.982 ± 2.746	0.004	0.270 ± .2761	0.360	0.854 ± 0.459	0.036
	III-IV	43	6.785 ± 2.740		0.221 ± 0.194		1.207 ± 0.965	

Grade	G1	30	4.835 ± 2.867	0.006	0.191 ± 0.132	0.123	0.802 ± 0.300	0.033
	G2-G3	50	6.621 ± 2.685		0.275 ± 0.276		1.189 ± 0.945	

Node status	Positive	23	4.834 ± 3.000	0.026	0.184 ± 0.140	0.149	1.317 ± 1.012	0.048
	Negative	57	6.402 ± 2.716		0.268 ± 0.261		0.933 ± 0.657	

Omentum metastasis	Yes	28	6.193 ± 2.712	0.584	0.172 ± 0.162	0.044	1.003 ± 0.595	0.734
	No	52	5.821 ± 2.971		0.283 ± 0.260		1.066 ± 0.880	

Organ metastasis^∗^	Yes	20	7.286 ± 2.397	0.015	0.181 ± 0.179	0.169	1.475 ± 1.072	0.032
	No	60	5.506 ± 2.896		0.265 ± 0.249		0.900 ± 0.616	

Ascites	≥500 ml	30	6.327 ± 2.697	0.367	0.186 ± 0.164	0.087	1.203 ± 1.047	0.227
	<500 ml	50	5.725 ± 2.975		0.279 ± 0.265		0.948 ± 0.574	

Platinum resistance	Resistant	28	4.804 ± 2.262	0.004	0.331 ± 0.295	0.014	1.333 ± 1.026	0.015
	Sensitive	52	6.569 ± 2.991		0.197 ± 0.183		0.888 ± 0.579	

Outcome	CR	27	7.272 ± 3.402	0.001	0.170 ± 0.085	0.032	0.793 ± 0.645	0.047
	PR	25	6.421 ± 2.434		0.224 ± 0.142		0.957 ± 0.630	
	SD	12	4.720 ± 1.960		0.268 ± 0.251		1.291 ± 0.546	
	PD	16	3.913 ± 1.384		0.383 ± 0.411		1.418 ± 1.174	

Notes: ^∗^: metastasis to any one of liver, lung, brain and spleen.

**Table 3:** Performance of FOLR1, DHFR, and MTRR in the differential diagnosis drug-resistant cases from ovarian cancer patients.

Groups	Sensitivity	Specificity	Accurate	Youden index	True positive	True negative	False positive	Flase negative
FOLR1	88.2%	55.2%	76.2%	43.4%	45	16	13	6
DHFR	69.0%	62.7%	65.0%	31.7%	20	32	19	9
MTRR	44.8%	92.2%	75.0%	37.0%	13	47	4	16
FOLR1+DHFR+MTRR	82.1%	84.6%	83.8%	66.8%	23	44	8	5

**Table 4:** The relation among FOLR1, DHFR, and MTRR expression in ovarian cancer.

Characteristics	FOLR1				DHFR			
	Positive case	Negative case	*κ* value	*P*	Positive case	Negative case	*κ* value	*P*
MTRR-positive case	11	6	30.189	0.001	8	9	11.025	0.001
MTRR- negative case	47	16			31	32		
DHFR-positive case	24	15	6.612	0.010	—	—	—	—
DHFR- negative case	34	7			—	—	—	—

**Table 5:** Univariate and multivariate analysis of survival in 80 patients with OC.

Variables	Univariate analysis			Multivariate analysis^a^		
	HR	95% CI	*P*	HR	95% CI	*P*
Histology						
Epithelial vs. other	1.690	0.798-3.852	0.171	—	—	—
FIGO Stage						
I-II vs. III-IV	2.349	1.143-4.826	0.020	1.004	0.319-3.164	0.994
Grade						
G1 vs. G2-G3	2.491	1.183-5.244	0.016	2.529	0.755-8.472	0.133
Node status						
Positive vs. negative	1.700	0.816-3.540	0.156	—	—	—
Omentum metastasis						
Positive vs. negative	0.868	0.428-1.761	0.695	—	—	—
Organ metastasis^∗^						
Positive vs. negative	1.168	0.560- 2.438	0.678	—	—	—
Ascites						
≥500 ml vs. <500 ml	2.175	1.072-4.411	0.031	1.715	0.489-6.016	0.400
Platinum resistance						
Resistant vs. sensitive	2.675	1.263-5.663	0.010	1.861	0.510-6.787	0.347
FOLR1-positive expression alone						
Positive vs. negative	0.824	0.394-1.721	0.606	—	—	—
DHFR-positive expression alone						
Positive vs. negative	1.410	0.720-2.761	0.316	—	—	—
MTRR-positive expression alone						
Positive vs. negative	2.334	1.066-5.110	0.034	1.444	0.607-3.432	0.406

Notes: ^a^Backward Wald test was used for variables screened. *P* < 0.05 was chosen as a criterion for significance. HR: hazard ratio; CI: 95% confidence interval. ^∗^Metastasis to any one of liver, lung, brain, and spleen.

## Data Availability

The data used to support the findings of this study are available from the corresponding author upon request.
